# Brief electrical stimulation and synkinesis after facial nerve crush injury: a randomized prospective animal study

**DOI:** 10.1186/s40463-018-0264-0

**Published:** 2018-03-07

**Authors:** Adrian Mendez, Alex Hopkins, Vincent L. Biron, Hadi Seikaly, Lin Fu Zhu, David W. J. Côté

**Affiliations:** 1 Department of Surgery, Division of Otolaryngology – Head and Neck Surgery, University of Alberta, Edmonton, AB Canada; 2 Faculty of Medicine and Dentistry, University of Alberta, Edmonton, AB Canada; 3 1E4 Walter C Mackenzie Centre, 8440-112 Street NW, Edmonton, AB T6G 2B7 Canada

**Keywords:** Synkinesis, Brief electrical stimulation, Facial nerve, Peripheral nerve regeneration, Regeneration, Peripheral nerve injury, Electrical stimulation

## Abstract

**Background:**

Recent studies have examined the effects of brief electrical stimulation (BES) on nerve regeneration, with some suggesting that BES accelerates facial nerve recovery. However, the facial nerve outcome measurement in these studies has not been precise or accurate. Furthermore, no previous studies have been able to demonstrate the effect of BES on synkinesis. The objective of this study is to examine the effect of brief electrical stimulation (BES) on facial nerve function and synkinesis in a rat model.

**Methods:**

Four groups of six rats underwent a facial nerve injury procedure. Group 1 and 2 underwent a crush injury at the main trunk of the nerve, with group 2 additionally receiving BES for 1 h. Group 3 and 4 underwent a transection injury at the main trunk, with group 4 additionally receiving BES for 1 h. A laser curtain model was used to measure amplitude of whisking at 2, 4, and 6 weeks. Fluorogold and fluororuby neurotracers were additionally injected into each facial nerve to measure synkinesis. Buccal and marginal mandibular branches of the facial nerve were each injected with different neurotracers at 3 months following injury. Based on facial nucleus motoneuron labelling of untreated rats, comparison was made to post-treatment animals to deduce whether synkinesis had taken place. All animals underwent trans-cardiac perfusion with subsequent neural tissue sectioning.

**Results:**

At week two, the amplitude observed for group 1 and 2 was 14.4 and 24.0 degrees, respectively (*p* = 0.0004). Group 4 also demonstrated improved whisking compared to group 3. Fluorescent neuroimaging labelling appear to confirm improved pathway specific regeneration with BES following facial nerve injury.

**Conclusions:**

This is the first study to use an implantable stimulator for serial BES following a crush injury in a validated animal model. Results suggest performing BES after facial nerve injury is associated with accelerated facial nerve function and improved facial nerve specific pathway regeneration in a rat model.

## Background

Facial neuromuscular disorders and functional impairment resulting from facial nerve injury are common and can be severe [1]. Aesthetic impairments also impart an affliction leading to social isolation and further emotional distress. Together these can lead to depressive symptoms and mental health issues, which further exacerbate their functional disabilities [2]. There are several clinical factors that have been identified that further impact recovery of peripheral nerve function following nerve injury including time to repair, type of repair, and the age of the patient [3].

Despite advances in microsurgical technique, functional recovery following facial nerve injury remains suboptimal [4]. Synkinesis, or axonal regeneration from the proximal stump into inappropriate distal pathways, has long been recognized as a significant contributing factor to poor functional recovery [5]. Previous studies have shown that electrical stimulation affects morphological and functional properties of neurons including nerve branching, rate and orientation of neurite growth, rapid sprouting, and guidance during axon regeneration [6, 7]. In 2010, Hadlock et al. studied the effect of electrical stimulation on the facial nerve in a rat model using a precise functional outcomes model capable of detecting micrometer movements of rat whisking [2]. The authors were able to demonstrate improvement in facial nerve functional outcome in the first 8 weeks. Similarly, in 2016 our research group published a study looking at the effect of BES on the transected facial nerve shortly after repair. We demonstrated improvement in facial nerve function with BES in the first 2 weeks after injury [8].

It has been hypothesized that the mechanism of action of BES is to induce preferential re-innervation of motor axons over sensory axons, and therefore improve overall function. In 2000, Gordon et al. examined the effect of electrical stimulation on regeneration after nerve transection in a rat sciatic nerve model [4]. The authors were able to demonstrate through retrograde labeling of sciatic nerve motoneurons with fluororuby (FR) and fluorogold (FG), that electrical stimulation dramatically accelerated both axonal regeneration as well as preferentially re-innervated motor nerves over sensory branches. The authors also found short-term, 1 h periods of stimulation were as effective as long-term stimulation lasting days to weeks [4].

Since then, the notion that brief electrical stimulation induces preferential re-innervation of motor axons over sensory axons has been extensively studied and is now well established. However, the effect of BES on reducing the random extension of specific motor axons collaterals to inappropriate distal motor axon branches such as in facial nerve synkinesis, is less clear.

Recently, research groups investigating peripheral nerve injury and regeneration have provided some insight into this question. Angelov and colleagues demonstrated that by using neutralizing antibodies to exogenous neurotrophic factors, including brain-derived neurotrophic factor (BDNF) and glial cell derived neurotrophic factor (GDNF), aberrant and redundant branching of regenerating axons in the facial nerve into inappropriate pathways could be reduced [9]. Furthermore, a separate research group demonstrated that BES is capable of regulation of BDNF expression in motoneurons [10]. Therefore, a possible mechanism of action of BES may be to reduce aberrant branching of regenerating motor axons following peripheral nerve injury by regulation BDNF expression in motoneurons. In regards to facial nerve injury and regeneration, this would potentially imply reduced synkinesis.

Furthermore, in 2005 Brushart et al. demonstrated that BES was capable of promoting the specific reinnervation of sensory pathways by the axotomized dorsal root ganglion sensory neurons [11]. This finding, which has since been replicated in other experimental designs, seems to indicate that BES is capable of not only preferential motor reinnervation, but overall pathway specific regeneration [12].

There are currently few studies that have examined the effect of BES in improving synkinesis of the facial nerve following injury. The primary objective of this study is to test the hypothesis that BES reduces synkinesis following facial nerve injury. A secondary objective is to examine the effect of BES on facial nerve function following injury.

## Methods

### Study design

This was a prospective randomized control animal trial conducted at the Surgical Medical Research Institute (SMRI) at the University of Alberta. Twenty-four rats were block randomized into four groups of six. Groups 1 and 2 underwent a crush injury at the main trunk of the nerve, with group 2 additionally receiving BES for 1 h. Groups 3 and 4 underwent a transection injury at the main trunk, with group 4 additionally receiving BES for 1 h. To investigate the effect of BES on synkinesis, the upper and lower main branches (buccal and marginal mandibular) of the facial nerve in all animals were back-labeled with two distinct neurotracers 3 months after injury. The brainstem of all animals was sectioned to identify the motoneurons supplying each of the two main branches. Comparison was made to a control motoneuron labeled brainstem.

To assess the effect of BES on function, facial nerve functional outcome assessment was collected at 2, 4, and 6 weeks post-operatively. A previously validated rat facial nerve model was used [13]. Ethics approval was obtained from the Animal Care and Use Committee (ACUC) overseen by the University Animal Policy and Welfare Committee (UAPWC) at the University of Alberta in Edmonton, Alberta [AUP00000785].

### Study subjects

Twenty four female Wistar rats (Charles River Laboratories, Canada) weighing 200–220 g were used as experimental animals for this study. Additional 2 control female Wistar rats were used. Sample size was calculated based on our previous study, which employed a similar outcome measure, powered to detect a difference of 10 degrees in whisking [13]. All rats were housed in pairs at the Health Sciences Laboratory Animal Services (HSLAS) at the University of Alberta. Rats were weighed and handled daily 2 weeks prior to the commencement of the study to reduce animal stress during the study.

### Facial nerve functional outcome assessment

The facial nerve functional outcome assessment model employed in this study was based on the model described and validated by Heaton et al. [13]. This model employs a head fixation device, body restraint, and bilateral photoelectric sensors to detect precise whisker movements as an objective measure for facial nerve function. The assessment model was set up and data was acquired using the methodology outlined in Mendez et al., 2016 [8].

### Data acquisition

Whisker movement was elicited in each subject by providing a scented stimulus (chocolate milk). The laser micrometers themselves were connected to a 32-Channel Digital I/O Module (NI 9403, National Instruments, Dallas, Tx), which received digital output from the laser micrometers. The I/O module was connected to a PC through a CompactDAQ chassis (cDAQ-9174, National Instruments, Dallas, Tx). The I/O module acquired the laser micrometer signal at a sampling rate of 1 kHz. LabVIEW (LabVIEW Full Development System, National Instruments, Dallas, Tx) software was used as the interface for data acquisition.

### Surgical procedure

All non-control subjects underwent both head implantation surgery as well as facial nerve surgery by a single surgeon during the same anesthetic. Groups 2 and 4 additionally received 1 h of BES following nerve injury while remaining anesthetized. All rats were first anesthetized with 3–4% isoflurane. Subjects were then maintained under general anesthesia using 1.5% isoflurane. Hair was then removed from the right side of the face and the top of the head using an electric shaver.

### Facial nerve surgery

All facial nerve surgery was completed on the right side of the face on all non-control subjects. A small incision was made just inferior to the right ear bony prominence. Under microscopic visualization, the parotid gland was visualized and everted and retracted out of the surgical field. Distal branches of the facial nerve were identified just inferior to the parotid bed. These were followed proximally until the bifurcation of the buccal and marginal mandibular branches of the facial nerve was identified. Once identified, the area proximal to the bifurcation of the facial nerve was carefully dissected. Groups 1 and 2 received a crush injury to the nerve. A hemostat instrument was applied across the facial nerve proximal to the bifurcation and clamped for a period of 30 s. Groups 3 and 4 received a transection injury to the nerve. A single, sharp transection of the facial nerve proximal to the bifurcation was made using straight microscopic scissors; the cut nerve ends were then immediately repaired using a direct end-to-end technique. Using 9–0 sutures, four simple interrupted sutures were made within the proximal and distal epineural nerve endings. Care was taken to ensure proper nerve alignment.

### Brief electrical stimulation

Along with facial nerve crush injury, animals in groups 2 and 4 received brief electrical stimulation. The protocol for stimulation was adapted from that used by Gordon et al. in the sciatic nerve rat model [4]. Two silver Teflon coated wires were bared of insulation for 2–3 mm (AGT0510, W-P Instruments, Inc.). Following nerve repair, the first wire was looped around the proximal stump of the facial nerve. The second wire was imbedded into muscle tissue adjacent to the facial nerve, at a location just proximal to the first wire. The insulated wires were led to a isostim stimulator (A320D, W-P Instruments, Inc.) which delivered a 1.5 mA current in pulses of 100 microseconds in a continuous 20 Hz train for a period of 1 h. The adequacy of stimulation was verified by the presence of a right ear flutter. At the completion of stimulation, the wires were removed from the animal and the incision closed with interrupted 3–0 vicryl sutures.

### Head implant surgery

Following the facial nerve procedure, head implant surgery was then completed without reversing the general anesthetic. A small incision was made using a 15-blade scalpel from the anterior to posterior margin of the cranium. Blunt dissection was employed to fully expose the underlying bony cranium. Using an electric drill, 4 holes were made in each quadrant of the skull approximately 15 mm apart from each other. 1.6 mm screws were then placed within each drill site. Dry acrylic resin was then liquefied and placed onto the skull, covering the placed screws. Two larger 5 mm threaded screws were then inverted with the threads directed upwards into the acrylic before it solidified.

### Head fixation and body restraint

**Fig. 1:**
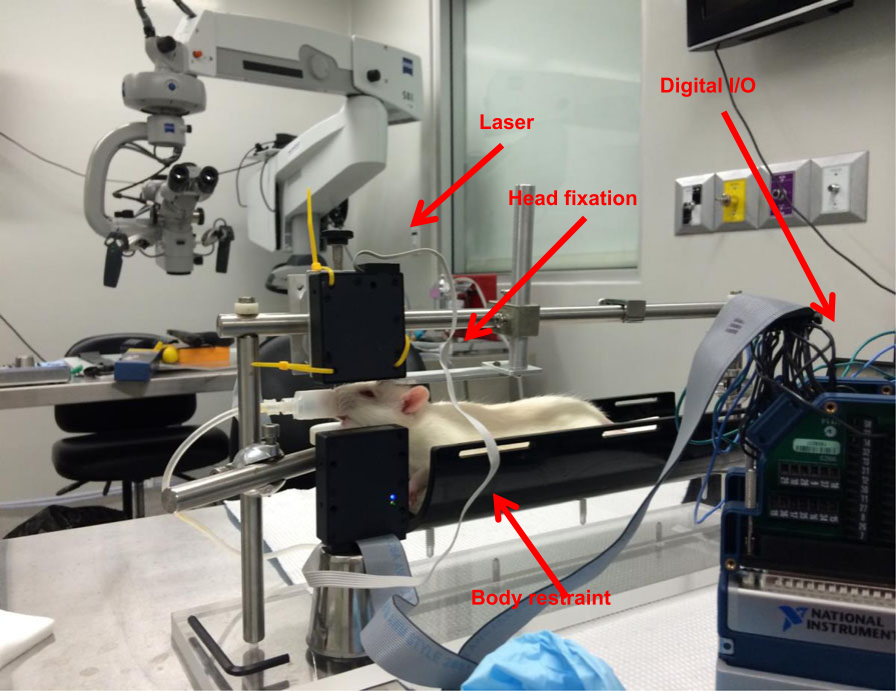
Whisking model

**Fig. 2:**
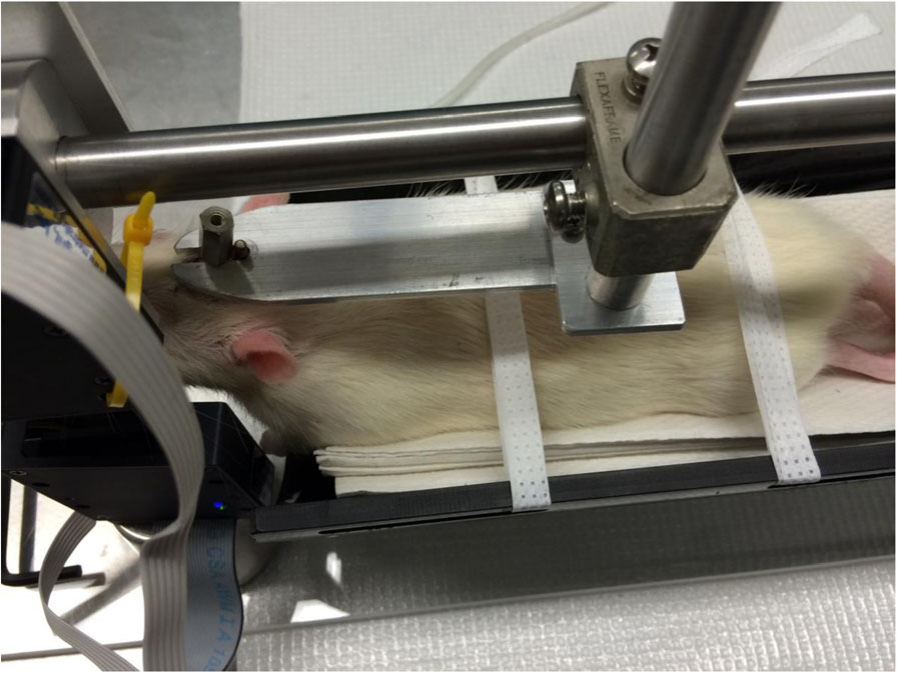
Head fixation

Two weeks prior to surgery, all animal subjects were handled daily for conditioning. After surgery, all subjects were placed in body restraints daily for a week. At post-operative day 14, whisker measurements were started. Subjects were initially given dose low dose isoflurane and transported to the body restraint apparatus (Fig. 1). Here they underwent head fixation with bolts applied across the exposed threaded screws (Fig. 2). Whisker markers were then placed on either side of the rat’s face.

Once this was completed, a scented stimulus was introduced and recording started usually for a period of 5 min. The non-operative left side was used as the control for each subject. This procedure was completed for each rat at two, four, and 6 weeks post-operatively.

### Retrograde labeling of motorneurons

At 3 months postoperatively, the buccal and marginal mandibular branches of the facial nerve were once again carefully dissected and identified. A timeline of 3 months following surgery was chosen as total nerve axonal regeneration is estimated to occur by 10 weeks following injury [4]. The buccal and marginal mandibular branches were then each sharply transected, 5 mm from the bifurcation. Each cut branch was then back-labeled with neurotracers to identify the motorneurons innervating each branch. FG and FR tracers were the neurotracers used, with each individual neurotracer labeling either the upper (buccal) or lower (marginal mandibular) branch.

Each neurotracer was first placed on a small piece of gelfoam. The gelfoam was then placed in contact with the cut end of the nerve branch for a period of 1 h. Each nerve branch was then copiously irrigated with saline. Care was taken to prevent cross labeling. Animals were kept for 4 days following neurotracer labeling to allow time for each neurotracer to reach the motorneurons in the brainstem.

### Tissue fixation by cardiac perfusion

Following neurotracer labeling, all animals underwent transcardiac perfusion in order to perform tissue fixation of the brainstem. Animals first had an intraperitoneal injection of ketamine. An intraabdominal incision was then made to expose the thorax, cardiac ventricles, and descending and ascending aorta. Using an 18 gage catheter, the left ventricle was penetrated and the catheter advanced until the tip was visualized in the ascending aorta. 300 mL of 1 M PBS was then perfused through the catheter. Following the PBS infusion, 400 mL of 4% Paraformaldehyde was then infused through the catheter. The animal was then decapitated and the entire brain exposed and removed. The brain specimen was placed in 4% Paraformaldehyde overnight and then switched over to 30% sucrose for 24 h. The tissue was then frozen in isopentane cooled at - 70 degrees Celsius and stored at - 80 degrees Celsius.

### Motoneuron counting

The frozen tissue specimens were removed from storage and sectioned in a cryostat at 20 μm coronal cuts. Sections were mounted on glass slides and dried. The sectioned brainstem cuts were then visualized using a fluorescent microscope with at 10× objective magnification under UV fluorescence at barrier filters of 580 nm for FR and 430 nanameters for FG. All motorneurons labeled with only FR (red), only FG (blue), or both were counted every sixth section. A blinded observer performed all counts and the counting of split cells was corrected for by the method of Abercrombie [14].

## Results

All animals tolerated the surgical procedure without perioperative complications. They exhibited normal cage behavior and did not lose weight.

### Functional outcome measurements

All experimental animals experienced complete ipsilateral loss of whisking amplitude post-operatively. At week two the average amplitude observed for group 1 (crush, no stimulation) was 14.4 degrees (Table 1). Showing a statistically significant improvement over group 1, the group 2 (crush with BES) average was 24.0 degrees at 2 weeks post-operatively (*p* = 0.0004). Group 3 (transection, no stimulation) and 4 (transection with BES) had average whisking amplitudes of 4.8 and 14.6 degrees, respectively, a statistically significant finding (Table 2). At week four, group 1 showed a minimal amplitude loss, with an average of 11.6 degrees, while group 2 remained relatively unchanged from week 2 with an average of 23.2 degrees. Group 3 and 4 exhibited average amplitudes of 9.1 and 13.0 degrees at week four, respectively. Group 1 had an average amplitude of 20.3 degrees at 6-weeks from surgery. Group 2 had an average amplitude of 26.7 degrees. There was no statistically significant difference between the two group 1 and 2 at 6 weeks after facial nerve surgery (*p* = 0.63). Group 3 and 4 recorded similar average amplitudes at 6 weeks of 13.4 and 15.2 degrees, respectively.

**Table 1:** Crush injury. Post-operative whisking amplitudes at week 2, 4, and 6

	Week 2 amplitude (degrees)	Week 4 amplitude (degrees)	Week 6 amplitude (degrees)
NERVE CRUSH (group 1) Right side (operated)	14.4	11.6	17.0
NERVE CRUSH (group 1) Left side (control)	69.7	73.3	67.2
NERVE CRUSH + BES (group 2) Right side (operated)	24.0	23.2	21.8
NERVE CRUSH + BES (group 2) Left side (control)	71.3	68.5	69.7
P value	0.0004	0.0002	0.6328

**Table 2:** Transection injury. Post-operative whisking amplitudes at week 2, 4, and 6

	Week 2 amplitude (degrees)	Week 4 amplitude (degrees)	Week 6 amplitude (degrees)
NERVE TRANSECTION (group 3) Right side (operated)	4.8	9.1	13.4
NERVE TRANSECTION (group 3) Left side (control)	72.1	66.6	71.8
NERVE TRANSECTION + BES (group 4) Right side (operated)	14.6	13.0	15.2
NERVE TRANSECTION + BES (group 4) Left side (control)	74.9	70.9	67.5
P value	0.0004	0.4715	0.5234

**Fig. 3:**
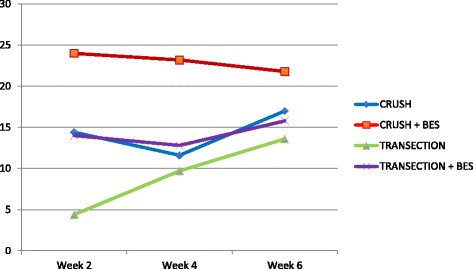
Whisking amplitude in degrees at 2, 4, and 6 weeks postoperatively. BES = brief electrical stimulation

Overall, BES significantly improved whisking capacity at two and 4 weeks post-injury in the animals that received a crush injury (*p* < 0.05). Similarly, BES significantly improved whisking capacity at 2 weeks post-injury in the animals that received a transection injury (p < 0.05). Finally, the BES crush injury animals (group 2) had statistically significant greater whisking capacity than the BES transection injury animals (group 4) at two, four, and 6 weeks post-injury (Fig. 3) (p < 0.05).

### Retrograde labeling of motorneurons

**Fig. 4:**
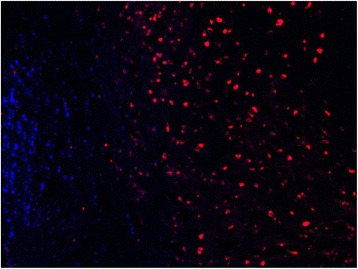
CONTROL - Facial nucleus after application of FR to the buccal branch and FG to marginal mandibular branch. FR = red, FG = blue. Note the myotopic organization of the nucleus

In the non-operated, control animals, a mean of 1388 fluoro-ruby (buccal branch) labeled motorneurons were counted, while a mean of 310 fluorogold (marginal mandibular) labelled motorneurons were observed (Table 3). No double labeled motorneurons were observed in the control animals. Visually, myotopic organization of the motorneurons was observed in the control animals (Fig. 4).

**Table 3:** Mean number of retrogradely labeled motorneurons, labeled either as only fluororuby, only fluorogold, or both

	Motorneurons labeled only with FR	Motorneurons labeled only with FG	Motorneurons labeled with FR + FG	Total labeled motorneurons
CONTROL	1488 (82%)	310 (17%)	25 (1%)	1823 (100%)
NERVE CRUSH (group 1)	723 (40%)	198 (11%)	889 (49%)	1810 (100%)
NERVE CRUSH with BES (group 2)	788 (43%)	209 (11%)	834 (46%)	1831 (100%)
NERVE TRANSECTION (group 3)	522 (27%)	88 (5%)	1299 (68%)	1909 (100%)
NERVE TRANSECTION with BES (group 4)	612 (31%)	126 (7%)	1222 (62%)	1960 (100%)

Group 1 and 2 had average counts of 989 (49%) and 934 (46%) double labeled motornuerons, respectively (*p* > 0.05). Group 3 and 4 had an average number of 1299 (68%) and 1222 (62%) double labeled motorneurons, respectively (p > 0.05). Both groups of animals that underwent BES (groups 2 and 4) had, on average, less double labeled motorneurons following facial nerve injury, than their non-stimulated counterpart (groups 1 and 3).

**Fig. 5:**
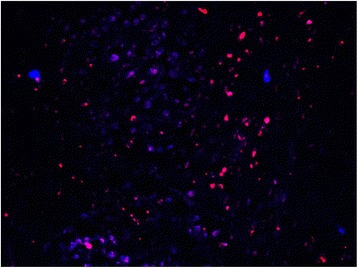
CRUSH + STIMULATION - Facial nucleus after application of FR to the buccal branch and FG to marginal mandibular branch. FR = red, FG = blue, double-labeled = pink. Note the decreased amount of myotopic organization

**Fig. 6:**
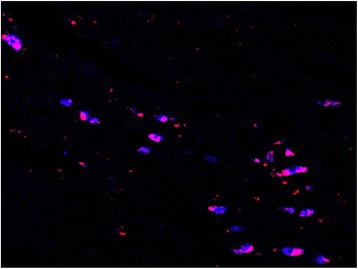
TRANSECTION - Facial nucleus after application of FR to the buccal branch and FG to marginal mandibular branch. FR = red, FG = blue, double-labeled = pink. Note the increased number of double labelled motorneurons

Overall, statistical significantly less double labeled motorneurons were analyzed in groups 1 and 2 (crush injury) as compared to groups 3 and 4 (transection injury) (*p* < 0.05). Groups 1 and 2 also displayed greater myotopic organization as compared to groups 3 and 4 (Figs. 5 and 6).

## Discussion

This study sought to evaluate the effect of brief electrical stimulation on synkinesis in a rat model for facial nerve injury. Through the retrograde examination of the facial nerve employing neurotracers, assessment was possible of the distribution of motor neurons in a control rat brainstem whose axons directly innervated either the buccal branch of the facial nerve branch or lower marginal mandibular branch. The buccal branch was labelled with fluroruby (FR) while flurogold (FG) was used to label the marginal mandibular branch of the facial nerve.

In the control animals, myotopic organization of the motorneurons was noted, with each motorneuron single labeled with either FR or FG(Fig. 4). In the experimental animals (groups 1 to 4), there was a significant increase in the number of double-labelled motoneurons (FR + FG) as well as a loss of myotopic organization of the facial motoneurons (Figs. 5 and 6).

These aberrant findings are thought to be caused by two principal processes present during peripheral nerve regeneration. The first process is malfunctioning axonal guidance, where an axon has been misguided along an incorrect fascicle [15]. In this study, this process likely affected the myotopic organization of the facial nucleus in the experimental animals. However, general comparison of the brainstem sections of the animals that had undergone crush injury (group 1, 2) as compared to those with a transection injury (groups 3, 4) revealed improved myotopic organization in the crush injury animals (Figs. 5 and 6). This finding was expected as crush injuries represent Sunderland level two injuries, which do not involve endoneurial disruption, while transection injuries represent a Sunderland level five injury. No appreciable difference in myotopic facial nucleus organization was noted between animals that received BES and those that did not.

The second principal process present during peripheral nerve regeneration is an increase in branches in all transected axons [16]. Because of this, following axonal injury a single motoneuron can send branches through numerous nerve fascicles. In our study, the presence of double-labelled motoneurons is likely due to this process, allowing a single motoneuron to re-innervate both the buccal and marginal mandibular branches, having deleterious effects on synchronized function. As expected, the crush injury animals (groups 1, 2) had significantly less percentage of double-labeled motoneurons as compared to the transection injury animals (groups 3, 4). Interestingly, the animals that received BES also had less percentage of double labelled motoneurons as compared to their non-BES counterparts. Although this finding was not statistically significant (*p* value), it does allude to the possibility that BES induces pathway specific regeneration. This would be in keeping with findings from other research groups.

This animal study also directly compared the facial nerve functional outcome in a group of rats receiving brief electrical stimulation following either crush or transection injury versus those not receiving stimulation. The results indicate a significant improvement in whisking amplitude in those animals receiving BES over those with the same injury that did not receive BES in the early weeks following nerve surgery. However, by week four and six post-operatively, no statistically significant difference seen between the two groups receiving transection or crush injuries, respectively. Results of this study are consistent with other reports investigating the effects of electrical stimulation on peripheral nerve regeneration [2, 4, 8]. Based on the neurotracer findings, a potential reason for the improved whisking function in the rats receiving BES is improved pathway specific regeneration of the facial nerve.

Gordon et al. have hypothesized that preferential motor reinnervation in a nerve injury model begins occurring at approximately 2 to 3 weeks following injury [4]. Up until that time, inappropriate sensory pathways are being created at the same rate as appropriate motor pathways. It appears that electrical stimulation is capable of starting preferential motor reinnervation at an earlier time point compared to non-stimulated nerves.

This is the first animal study incorporating neurotracer retrograde labeling of the facial nerve and brief electrical stimulation. The results of this study taken together with the findings of other researchers indicate the potential for acceleration of facial nerve function with electrical stimulation in animals. Interestingly, BES may also induce pathway specific regeneration of motoneurons following facial nerve injury. Although there are currently no human trials using BES following facial nerve injury, its application in the human clinical setting appears promising.

## Conclusion

This study demonstrates brief electrical stimulation of a rat facial nerve crush injury model is associated with accelerated facial nerve functional outcome. BES may also be capable of inducing pathway specific regeneration of motoneurons following facial nerve injury. This has interesting clinical benefits and potential applications in human facial nerve injuries.

## Abbreviations

ACUC: Animal care and use committee
BDNF: Brain-derived neurotrophic factor
BES: Brief electrical stimulation
FG: Fluorogold
FR: Fluororuby
GDNR: Glial cell derived neurotrophic factor
HSLAS: Health sciences laboratory animal services
UAPWC: University animal policy and welfare committee
